# Helichrysum Essential Oil Ameliorates Atopic Dermatitis by Suppressing the NLRP3 Inflammasome and Restoring Epidermal Barrier Integrity

**DOI:** 10.3390/cimb48100996

**Published:** 2026-09-26

**Authors:** Yongqi Chen, Zhongying Xu, Liming Mao, Congcong Shen

**Affiliations:** 1 Department of Dermatology, Affiliated Hospital of Nantong University, Medical School of Nantong University, 20 Xisi Road, Nantong 226001, China; 2431320326@stmail.ntu.edu.cn (Y.C.); 2531320373@stmail.ntu.edu.cn (Z.X.); 2 Department of Immunology, Medical School of Nantong University, 19 Qixiu Road, Nantong 226001, China; 3 Basic Medical Research Center, Medical School of Nantong University, 19 Qixiu Road, Nantong 226019, China

**Keywords:** Atopic Dermatitis, *Helichrysum* essential oil, inflammatory mediators, NLRP3 inflammasome, skin barrier

## Abstract

Atopic dermatitis (AD) is a chronic inflammatory skin disease characterized by immune dysregulation and epidermal barrier dysfunction. The essential oil of *Helichrysum* (HEO) possesses known anti-inflammatory properties, yet its therapeutic efficacy and underlying mechanisms in AD remain to be fully elucidated. This study aimed to investigate the protective effects of HEO on AD-like skin lesions and to explore its regulatory mechanisms involving the NLRP3 inflammasome and skin barrier proteins. Lipopolysaccharide (LPS)-stimulated HaCaT keratinocytes and RAW264.7 macrophages were treated with HEO to assess cell viability, nitric oxide (NO) production, and the expression of inflammatory mediators (*COX-2*, *iNOS*, *IL-1β*, *NLRP3*). For in vivo studies, an AD mouse model was induced by repeated application of 1-chloro-2,4-dinitrobenzene (DNCB). Mice were treated topically with HEO, and skin severity scores, ear thickness, and histological changes were evaluated. Expression levels of inflammatory markers and barrier proteins were analyzed via Western blotting and qRT-PCR. Serum levels of cytokines and liver function markers were also measured. Our results showed that, using DNCB-induced AD mouse model, topical application of HEO markedly attenuated dermatitis severity, reduced ear thickness, and suppressed epidermal hyperplasia. Furthermore, HEO treatment alleviated systemic inflammation and liver dysfunction, as evidenced by reduced serum levels of TNF-α, IL-6, AST, ALT, and ALP. Mechanistically, HEO significantly inhibited NO production and downregulated the mRNA and protein expression of COX-2, iNOS, and the NLRP3/IL-1β axis in LPS-stimulated cells. Moreover, it effectively inhibited the activation of NLRP3 inflammasome and altered the expression of skin barrier proteins, Filaggrin and Loricrin, in dorsal tissues of DNCB-treated mice. Together, these data demonstrated that HEO ameliorates AD-like symptoms by suppressing the NLRP3 inflammasome-mediated inflammatory response and restoring epidermal barrier integrity. These findings suggest that HEO is a promising therapeutic candidate for the development of drugs for AD.

## 1. Introduction

Atopic Dermatitis (AD) is a prevalent, chronic, relapsing inflammatory skin disorder characterized by intense pruritus, xerosis, and epidermal barrier dysfunction [1]. Over the past few decades, the global burden of AD has increased markedly. Current epidemiological data indicate that AD affects approximately 15~20% of children and 1~10% of adults worldwide, with substantial regional variation [2]. Prevalence is particularly elevated in industrialized countries, posing a significant public health challenge and profoundly impairing patients’ quality of life through both physical discomfort and psychological morbidity. Current first-line therapies rely predominantly on topical corticosteroids (TCS) and topical calcineurin inhibitors (TCIs) to suppress cutaneous inflammation [3]. However, long-term disease control remains limited by safety concerns. Prolonged TCS use is associated with well-documented cutaneous adverse effects, including skin atrophy, striae, and telangiectasia, as well as potential systemic absorption leading to hypothalamic–pituitary–adrenal (HPA) axis suppression [4]. TCIs, while steroid-sparing, carry FDA-mandated black-box warnings regarding theoretical risks of immunosuppression and malignancy, albeit with no conclusive epidemiological evidence to date [5]. These constraints underscore an unmet clinical need for safer, equally efficacious therapeutic alternatives.

The pathogenesis of AD involves a dynamic triad: immune dysregulation, epidermal barrier disruption, and environmental exposure. Central to this framework is the “outside–inside–outside” hypothesis. Immune activation constitutes a core driver: activated keratinocytes and infiltrating immune cells [6], including Th2 lymphocytes and macrophages, secrete a broad spectrum of inflammatory mediators, such as IL-4, IL-13, IL-1β, chemokines, COX-2, and iNOS. Critically, the NOD-like receptor protein 3 (NLRP3) inflammasome serves as a key molecular sensor; its activation triggers caspase-1–dependent maturation and release of IL-1β and IL-18, thereby amplifying inflammation and further compromising barrier integrity [7]. Conversely, genetic or acquired deficiencies in structural barrier proteins, particularly filaggrin (FLG) and loricrin (LOR), increase stratum corneum permeability. This permits penetration of allergens, pollutants, and microbial components, which activate keratinocytes to release alarmins (such as TSLP and IL-33), initiating and sustaining a maladaptive cutaneous immune response [8]. Thus, inflammation and barrier failure engage in a self-perpetuating, bidirectional loop that underlies AD chronicity.

Given these limitations of conventional therapeutics, phytochemical interventions have garnered increasing scientific interest. *Helichrysum* species have been used for centuries in traditional medicine across Mediterranean and African regions, and *Helichrysum* essential oil (HEO) is widely recognized for its antioxidant, antimicrobial, and anti-inflammatory properties [9]. Prior preclinical studies report that HEO and its principal bioactive constituents, including italidiones and neryl acetate, exert anti-inflammatory effects in diverse models. For example, HEO accelerates wound healing by promoting M2 macrophage polarization and attenuating oxidative stress [10]. Moreover, *Helichrysum* extracts inhibit key pro-inflammatory enzymes, including 5-lipoxygenase and cyclooxygenase, in vitro and in animal models, suggesting mechanistic relevance to dermatitis pathophysiology [11,12]. Nevertheless, direct evidence for HEO’s efficacy in AD, and its specific modulatory effects on the NLRP3 inflammasome pathway and epidermal barrier protein expression, remains lacking.

In this study, we systematically evaluated the therapeutic potential of HEO in AD using both in vitro and in vivo models. We employed LPS-stimulated HaCaT keratinocytes and RAW264.7 macrophages to dissect anti-inflammatory mechanisms at the cellular level, and a DNCB-induced murine AD model to assess translational efficacy in vivo. Our results demonstrate that HEO treatment significantly ameliorates AD-like phenotypes by concurrently suppressing the NLRP3/IL-1β signaling axis and restoring the expression of critical barrier proteins such as filaggrin and loricrin. Collectively, these findings provide robust, mechanism-based experimental evidence supporting HEO as a promising, safe, and biologically rational candidate for AD management, thereby bridging ethnobotanical tradition with contemporary molecular dermatology.

## 2. Materials and Methods

### 2.1. Reagents

HEO was purchased from POLI Aromatic Medicine Technology (Shanghai, China) Co., Ltd. (Item No.:OEI0101, Country of Origin: Bosnia, Herzegovina). It was extracted from *Helichrysum italicum* (Roth) G.Don. The oil was stored in amber vials at 4 °C to protect it from light and oxidation until use. For experimental procedures, HEO was dissolved in dimethyl sulfoxide (DMSO) to prepare a stock solution and subsequently diluted with culture medium to the required concentrations immediately prior to use. The final concentration of DMSO in the culture medium was kept below 0.1% (*v*/*v*) to avoid cytotoxicity. LPS was purchased from InvivoGen (Cat# tlrl-eb, San Diego, CA, USA).

### 2.2. Cell Culture and Treatment

Human immortalized keratinocytes (HaCaT cells, KCB200442YJ) and murine macrophage cells (RAW264.7, ATCC TIB-71) were obtained from Cell Bank of Type Culture Collection of the Chinese Academy of Sciences (Shanghai, China). Cells were cultured in Dulbecco’s Modified Eagle Medium (DMEM) supplemented with 10% fetal bovine serum (FBS) and 1% penicillin-streptomycin at 37 °C in a humidified atmosphere containing 5% CO_2_. For inflammation induction, cells were stimulated with lipopolysaccharide (LPS, 1 μg/mL) for 24 h after pretreatment with various concentrations of HEO (1, 5, 10, 20, and 50 μg/mL) for 2 h.

### 2.3. Cell Viability Assay

The cytotoxicity of HEO was assessed using the Cell Counting Kit-8 (CCK-8, Cat# C0042, Beyotime Biotechnology, Shanghai, China) assay. HaCaT cells were seeded in 96-well plates at a density of 1 × 10^4^ cells/well and treated with HEO at concentrations ranging from 0 to 100 μg/mL for 24 h, both in the presence and absence of LPS (1 μg/mL). The optical density was measured at 450 nm using a microplate reader. Cell viability was calculated as a percentage relative to the untreated control group.

### 2.4. Quantitative Real-Time PCR (qRT-PCR)

Total RNA was extracted from HaCaT and RAW264.7 cells using TRIzol reagent (Cat# R0016, Beyotime Biotechnology, Shanghai, China). Complementary DNA (cDNA) was synthesized using a PrimeScript RT reagent kit (Cat# RR037A, Takara Bio, Otsu, Shiga, Japan). The mRNA expression levels of COX2, iNOS, NLRP3, and IL-1β were quantified using SYBR Green Master Mix (Cat# D7260, Beyotime Biotechnology, Shanghai, China) on a real-time PCR detection system. The relative gene expression was normalized to the internal control (GAPDH or β-actin) and calculated using the 2^(-ΔΔCt) method.

### 2.5. Western Blot Analysis

Cells and dorsal skin tissues were lysed using RIPA buffer (P0039, Beyotime Biotechnology, Shanghai, China) containing protease (11836153001, Roche, Basel, Switzerland) and phosphatase inhibitors (04906837001, Roche, Basel, Switzerland). Protein concentrations were determined using a BCA assay. Equal amounts of protein were separated by SDS-PAGE and transferred onto polyvinylidene difluoride (PVDF) membranes. Membranes were pre-incubated with 5% non-fat dry milk in Tris-buffered saline containing 0.1% Tween-20 (TBST) to block nonspecific binding sites, followed by overnight incubation at 4 °C with primary antibodies targeting COX-2 (Cat. #12282, Cell Signaling Technology, Danvers, MA, USA), iNOS (Cat. #13120, Cell Signaling Technology, Danvers, MA, USA), IL-1β (Cat. #12703, Cell Signaling Technology, Danvers, MA, USA), filaggrin (Cat. #27156, Cell Signaling Technology, Danvers, MA, USA), loricrin (Cat. #15638, Cell Signaling Technology, Danvers, MA, USA), and β-actin (Cat. #4967, Cell Signaling Technology, Danvers, MA, USA). All antibodies were diluted in blocking buffer according to the manufacturer’s specifications. After incubation with horseradish peroxidase (HRP)-conjugated secondary antibodies, protein bands were visualized using an enhanced chemiluminescence (ECL) detection system. Band intensities were quantified using ImageJ software (version 1.54f; National Institutes of Health, Bethesda, MD, USA).

### 2.6. Nitric Oxide (NO) Production Assay

The accumulation of nitrite in the culture supernatants of RAW264.7 cells was measured as an indicator of NO production. After treatment with HEO and LPS, the supernatants were collected and mixed with Griess reagent (Cat# G2930, Promega Corporation, Madison, WI, USA). The absorbance was measured at 540 nm, and the nitrite concentration was calculated based on a standard curve generated with sodium nitrite.

### 2.7. Animal Model of AD

Female BALB/c mice (6–8 weeks old, weighing 18–22 g) were purchased and acclimatized for one week under specific pathogen-free conditions. A total of 42 mice were randomly assigned to seven experimental groups (*n* = 6 per group) using a computer-generated random number sequence (Excel RAND function): (1) Sham group (normal control without DNCB challenge, treated with vehicle only), (2) HEO-only group (normal mice treated with 5% HEO without DNCB challenge), (3) Con group (DNCB-challenged mice treated with vehicle), (4–6) DNCB-challenged mice treated with HEO at 3%, 5%, or 10%, and (7) DNCB-challenged mice treated with Dexamethasone (Dexa) as positive drug control. AD was induced by repeated application of 1-chloro-2,4-dinitrobenzene (DNCB, Cat# D198501, Sigma-Aldrich, St. Louis, MO, USA) on both dorsal skin and ears according to established protocols with slight modifications [13]. Following depilation of the dorsal skin on day 0, mice received topical application of 100 μL of 3% DNCB on the dorsal skin and 20 μL on the ears (10 μL per ear) on days 1, 4, and 7 for sensitization. From day 13 to day 20, mice were challenged with 0.5% DNCB every three days (50 μL on dorsal skin, 10 μL per ear) to induce chronic AD-like lesions, and concurrently received daily topical treatment with HEO (3%, 5%, or 10%), Dexa, or vehicle applied separately to the dorsal skin (50 μL) and ears (10 μL per ear). On day 21, all mice were euthanized in a CO_2_ chamber, and blood and spleen samples were collected. Dorsal skin was excised, with portions fixed in 4% paraformaldehyde for histological analysis, embedded in optimal cutting temperature compound for sectioning, or snap-frozen at −80 °C for molecular analyses.

To minimize the confounding effects of inter-individual biological variation inherent in animal skin tissues, we employed a sample-pooling strategy for sample preparation for qRT-PCR and Western blot analysis. Specifically, the six mice in each experimental group were randomly divided into three subgroups, each containing two mice. Skin tissues from the two mice within each subgroup were pooled together prior to protein extraction. Consequently, the *n* = 3 indicated in the Western blot figures represents three independent biological replicates (each derived from a pooled sample of two mice). This approach ensured high-quality protein yields and enhanced the reliability of our quantification by reducing individual outliers.

Mice were housed in a specific pathogen-free facility at 22 ± 2 °C with 50 ± 10% relative humidity under a 12-h light/dark cycle, group-housed (4–5 per cage) in individually ventilated cages with sterile corncob bedding, nesting material, and cardboard tubes as environmental enrichment, with ad libitum access to standard rodent chow and autoclaved water. Only female BALB/c mice aged 6–8 weeks without visible skin lesions or signs of illness after acclimatization were included; mice showing infection, injury, or abnormal behaviour were excluded a priori, and no animals were excluded after randomisation. All procedures were designed to minimize pain and distress, with mice monitored daily by trained staff for excessive scratching, weight loss exceeding 15%, hunched posture, ruffled fur, or reduced activity. Humane endpoints comprised body weight loss greater than 20%, severe skin ulceration exceeding 10% of the treated area, persistent pain unresponsive to analgesia, or inability to access food or water; no animals reached these endpoints or experienced unexpected adverse events, though mild transient erythema at application sites was observed in DNCB-challenged mice as expected. Investigators performing outcome assessment and data analysis were blinded to group allocation throughout the experiment, with group identities concealed using coded labels, although treatment application could not be fully blinded due to the distinct odour of HEO. Sample size was determined based on prior experience with the DNCB-induced AD model and consistent with previous publications using similar experimental designs [13], without formal a priori power calculation. The study protocol was prepared and approved by the Institutional Animal Care and Use Committee of Nantong University (Approval No. S20250812-011, 12 August 2025).

### 2.8. GC-MS Analysis

The chemical composition of HEO was analyzed by gas chromatography-mass spectrometry (GC-MS) using an Agilent 7890B gas chromatograph coupled to an Agilent 5977B mass selective detector (Agilent Technologies, Santa Clara, CA, USA). The essential oil was extracted by hydrodistillation from the aerial parts of H. italicum and stored in amber vials at 4 °C until analysis. Prior to injection, HEO was diluted 1:100 (*v*/*v*) with n-hexane (HPLC grade), and 1 μL of the diluted sample was injected in split mode (split ratio 1:50). Separation was performed on a non-polar HP-5MS fused-silica capillary column (30 m × 0.25 mm i.d., 0.25 μm film thickness; Agilent Technologies). Helium (99.999% purity) was used as the carrier gas at a constant flow rate of 1.0 mL/min. The oven temperature program was as follows: initial temperature 50 °C held for 2 min, ramped to 250 °C at a rate of 5 °C/min, and held at 250 °C for 10 min. The injector temperature was set at 250 °C, and the transfer line temperature was maintained at 280 °C. Mass spectra were acquired in electron ionization (EI) mode at 70 eV, with a scan range of m/z 50–500 and a solvent delay of 3 min. The ion source temperature was set at 230 °C. Constituents were identified by comparing their mass spectra with the NIST 3.0 mass spectral library and by matching retention times with reference standards. The relative percentage of each identified compound was calculated from the peak area normalization method without applying response factors.

### 2.9. Statistical Analysis

Data are presented as mean ± standard deviation (SD). Statistical differences between groups were analyzed using one-way analysis of variance (ANOVA) followed by Tukey’s post-hoc test for multiple comparisons. A value of *p* < 0.05 was considered statistically significant.

## 3. Results

### 3.1. GC-MS Analysis of HEO

HEO is a yellowish liquid with a characteristic woody and spicy aroma. It is obtained from the aerial parts of *H. italicum*. The chemical composition of HEO utilized in this study was analyzed by GC-MS, and the total ion chromatogram (TIC) is presented in Figure 1. A total of 32 compounds were identified, accounting for 99.42% of the total oil composition (Table 1).

The major components of HEO include longipinene (17.60%), 1,3,3-trimethyltricyclo [2.2.1.0^2^,^6^]heptane (15.42%), β-caryophyllene (10.67%), (Z)-3,7-dimethyl-2,6-octadien-1-ol acetate (neryl acetate, 9.35%), β-selinene (7.76%), and α-selinene (5.37%). Other notable constituents include copaene (4.68%), (+)-limonene (4.02%), and cyclopenta [1,4]cyclobuta [1,2]benzene derivative (5.85%).

Chemically, HEO was characterized by a high content of sesquiterpene hydrocarbons (45.18%), followed by oxygenated monoterpenes (14.23%), monoterpene hydrocarbons (6.51%), and oxygenated sesquiterpenes (5.85%). The predominant presence of longipinene and β-caryophyllene distinguishes this chemotype from the typical neryl acetate-rich chemotype reported for *Mediterranean H. italicum* populations, suggesting a unique chemical profile potentially influenced by geographical origin or environmental conditions.

### 3.2. Effect of HEO on the Cell Viability of Keratinocytes and Macrophages

To evaluate the cytotoxic potential of HEO, cell viability was assessed in human immortalized keratinocyte (HaCaT) cells and murine macrophage (RAW264.7) cells under both unstimulated and lipopolysaccharide (LPS)-activated conditions. As illustrated in Figure 2, HEO exhibited minimal cytotoxicity at concentrations lower than 20 μg/mL across all tested cell types. In HaCaT cells under basal conditions (Figure 2A), cell viability remained comparable to the untreated control at concentrations ranging from 0 to 20 μg/mL. A statistically significant reduction in viability was first observed at 50 μg/mL (*p* < 0.01), with a more pronounced dose-dependent decline at 100 μg/mL (*p* < 0.001). Similarly, in LPS-stimulated HaCaT cells (Figure 2B), viability was well-preserved at concentrations up to 20 μg/mL. Significant cytotoxicity emerged at higher concentrations, with marked reductions observed at 50 and 100 μg/mL (*p* < 0.001). Parallel results were observed in RAW264.7 macrophages. Under basal conditions (Figure 2C), HEO treatment did not significantly affect cell viability up to 20 μg/mL. However, concentrations of 50 μg/mL and above induced a significant, dose-dependent decrease in viability (*p* < 0.001 for 50~100 μg/mL). In LPS-stimulated RAW264.7 cells (Figure 2D), a significant reduction was noted at 50 μg/mL (*p* < 0.05), followed by severe cytotoxicity at 100 μg/mL (*p* < 0.001). Collectively, these results demonstrate that HEO maintains high cell viability in both keratinocytes and macrophages at concentrations ≤ 20 μg/mL, establishing this range as safe for downstream functional assays. Concentrations of 20 μg/mL and above begin to show varying degrees of cytotoxicity depending on the cell type and stimulation status.

### 3.3. HEO Suppresses LPS-Induced Inflammatory Gene Expression in Keratinocytes and Macrophages

We further explored the potential roles of HEO in inflammatory responses by examining its impact on the gene expression of crucial pro-inflammatory mediators in HaCaT and RAW264.7 cells stimulated with LPS. First, we assessed the expression of iNOS, COX2, NLRP3, and IL-1β in murine macrophage RAW264.7 cells (Figure 3A–D). Compared to the unstimulated control, LPS stimulation significantly upregulated the mRNA levels of all four inflammatory markers. Treatment with HEO (1, 5, 10, and 20 μg/mL) led to a dose-dependent suppression of these genes. Consistent with these results, we observed similar inhibitory effects in human keratinocyte HaCaT cells (Figure 3E–H). LPS induced a substantial increase in the transcription of iNOS, COX2, NLRP3, and IL-1β. Co-treatment with HEO significantly mitigated this LPS-induced upregulation in a concentration-dependent manner. Collectively, these data indicate that HEO effectively inhibits the transcriptional activation of major pro-inflammatory factors and inflammasome components in both macrophages and keratinocytes.

To further verify the anti-inflammatory effects of HEO at the protein level, Western blot analysis was conducted to detect the expression of COX2 and iNOS in LPS-stimulated HaCaT and RAW264.7 cells. As depicted in Figure 4A and Supplementary Materials, compared with the untreated control group, stimulation with LPS (1 μg/mL) significantly upregulated the protein levels of both iNOS and COX2 in HaCaT cells. Pretreatment with HEO (5, 10, and 20 μg/mL) notably suppressed this LPS-induced protein expression in a dose-dependent manner. Quantitative analysis corroborated that the relative protein levels of iNOS and COX2 decreased gradually as the concentration of HEO increased, with the 20 μg/mL group showing the most significant reduction (*p* < 0.001). Likewise, in RAW264.7 macrophages (Figure 4B and Supplementary Materials), LPS stimulation resulted in a substantial increase in COX2 and iNOS protein expression. Treatment with HEO at concentrations ranging from 5 to 20 μg/mL effectively curbed the production of these inflammatory mediators. The inhibitory effect was also concentration-dependent, with the highest concentration of HEO (20 μg/mL) exhibiting the most potent suppression of both COX2 and iNOS protein levels. These findings indicate that HEO not only downregulates the transcription but also significantly inhibits the translation of key pro-inflammatory enzymes, COX2 and iNOS, in both keratinocytes and macrophages.

Given that iNOS is the key enzyme responsible for nitric oxide (NO) synthesis during inflammation, we further investigated whether the downregulation of iNOS by HEO translates to reduced NO production. As shown in Figure 5A, stimulation of HaCaT cells with LPS resulted in a dramatic increase in nitrite accumulation in the culture medium compared to the unstimulated control. Treatment with HEO significantly attenuated this LPS-induced NO production in a concentration-dependent manner. Specifically, HEO concentrations of 5, 10, 20, and 50 μg/mL progressively reduced nitrite levels, with the highest inhibition observed at 50 μg/mL. Similarly, in RAW264.7 macrophages (Figure 5B), LPS stimulation induced a robust release of nitrite. Co-treatment with HEO effectively suppressed this response. Consistent with the results in HaCaT cells, HEO exhibited a dose-dependent inhibitory effect on NO production in macrophages, with significant reductions observed at all tested concentrations (5–50 μg/mL). These findings confirm that HEO functionally inhibits the enzymatic activity or expression of iNOS, thereby reducing the release of the pro-inflammatory mediator NO in both cell types.

### 3.4. HEO Significantly Alleviates DNCB-Induced AD in Mice

To evaluate the in vivo therapeutic efficacy of HEO, a DNCB-induced AD mouse model was established. As illustrated in the experimental timeline (Figure 6A), mice were sensitized with 1% DNCB and subsequently challenged with 0.5% DNCB. Topical treatment with varying concentrations of HEO (3%, 5%, and 10%) or the positive control drug Dexa was administered from day 13 to day 20. Macroscopic examination of the dorsal skin and ears revealed that repeated application of DNCB induced severe AD-like symptoms, characterized by erythema, edema, excoriation, and dryness compared to the normal control group (Figure 6B). However, topical application of HEO markedly alleviated these clinical symptoms. Among the treatment groups, the 5% HEO treatment group showed most substantial improvement in skin lesions, characterized by reduced redness and swelling, comparable to the effects observed in the Dexa-treated group. Consistent with the visual observations, quantitative measurement of ear thickness, a key indicator of edema and inflammation, demonstrated that DNCB challenge (Con group) significantly increased ear thickness compared to the untreated control (Figure 6C). While all HEO concentrations significantly alleviated ear swelling, the 5% group displayed the strongest therapeutic potential in terms of mean values. The lack of statistical distinction between doses suggests a non-linear dose–response relationship. Furthermore, histopathological analysis of the skin lesions via H&E staining was performed to assess tissue-level changes (Figure 6D). The skin sections from the DNCB group exhibited typical AD pathological features, including significant epidermal hyperplasia, hyperkeratosis, and massive infiltration of inflammatory cells into the dermis. In contrast, HEO treatment markedly attenuated these histological alterations. The epidermal thickness was reduced, and inflammatory cell infiltration was visibly decreased in the HEO-treated groups, particularly at the 10% concentration, which showed efficacy similar to Dexa. These findings collectively suggest that HEO possesses potent anti-inflammatory activity and effectively alleviates both macroscopic and microscopic symptoms of DNCB-induced skin inflammation in vivo.

### 3.5. HEO Suppresses the Expression of Inflammatory Mediators and Alarmins in DNCB-Induced AD Skin Lesions

To further elucidate the molecular mechanisms underlying the therapeutic effects of HEO on AD, we analyzed the mRNA expression levels of key pro-inflammatory mediators and epithelial-derived alarmins in dorsal skin tissues using qRT-PCR. Given that AD pathogenesis is initiated by alarmins such as TSLP and IL-33 [14], we first assessed their expression to validate the model and treatment efficacy. As shown in Figure 7A,B, DNCB challenge significantly upregulated the transcription of TSLP and IL-33 in the Con group compared to the Sham group. Topical application of HEO effectively reversed these changes. Notably, all tested concentrations of HEO (3%, 5%, and 10%) significantly reduced the mRNA levels of both TSLP and IL-33 (*p* < 0.001). Among these, the 5% HEO group displayed the strongest therapeutic potential in terms of mean values, demonstrating a potent suppression of these upstream Th2-polarizing signals.

Furthermore, we examined the associated innate inflammatory pathways. DNCB challenge significantly increased the transcription of iNOS, Cox2, NLRP3, and IL-1β (Figure 7C–F). Treatment with HEO suppressed the expression of these markers. Specifically, 5% and 10% HEO significantly reduced the mRNA levels of all four inflammatory markers compared to the Con group (*p* < 0.01 or *p* < 0.001). Notably, the inhibitory effect of 5% HEO on iNOS, Cox2, NLRP3, and IL-1β expression was most pronounced in terms of mean values. These results suggest that HEO alleviates AD-like skin inflammation by suppressing both the epithelial alarmin signaling (TSLP/IL-33) and the activation of the iNOS/COX2 pathway and NLRP3 inflammasome in vivo.

### 3.6. HEO Ameliorates Systemic Inflammation and Liver Dysfunction in DNCB-Induced Mice

To investigate whether the topical application of HEO exerts systemic effects beyond the skin lesions, we analyzed the levels of pro-inflammatory cytokines and liver function markers in the serum. As shown in Figure 8A,B, DNCB induction led to a significant elevation in serum levels of TNF-α and IL-6 compared to the control group, indicating a systemic inflammatory response. Treatment with HEO (DNCB + HEO group) significantly suppressed the secretion of both cytokines, suggesting an alleviation of systemic inflammation. Furthermore, we assessed potential liver toxicity or dysfunction associated with the AD model and treatment. The DNCB group exhibited markedly elevated levels of liver injury markers, including aspartate aminotransferase (AST), alanine aminotransferase (ALT), and alkaline phosphatase (ALP) (Figure 8C–E), alongside a significant decrease in albumin (ALB) levels (Figure 8F). Notably, HEO treatment effectively reversed these abnormalities: it significantly reduced the elevated AST, ALT, and ALP levels while restoring ALB levels closer to those of the control group. These findings indicate that HEO not only mitigates local skin inflammation but improves systemic inflammatory status and protects against liver dysfunction in DNCB-treated mice.

### 3.7. HEO Modulates the Expression of Skin Barrier Proteins and Inhibits NLRP3 Inflammasome Activation and IL-1β Maturation in DNCB-Induced AD Mice

The integrity of the epidermal barrier is critical in the pathogenesis of AD. We therefore investigated the effect of HEO on the protein expression of key structural components, Loricrin (LOR) and Filaggrin (FLG), in dorsal skin tissues via Western blot analysis (Figure 9A and Supplementary Materials). As quantified in Figure 9B, DNCB challenge significantly downregulated the protein level of FLG compared to the Sham group, indicating barrier disruption. Topical application of HEO effectively restored FLG expression. Notably, the 5% HEO treatment exhibited the most potent restorative effect (*p* < 0.001), significantly upregulating FLG levels compared to the Con group. Regarding LOR expression (Figure 9C), the Con group exhibited a significantly reduced level compared to the Sham group (*p* < 0.001). While treatment with HEO at concentrations of 3%, 5%, and 10% did not restore LOR expression, instead, it significantly reduced LOR levels relative to the Con group. In contrast, the positive control Dexa failed to restore the expression of either FLG or LOR. Collectively, these results indicate that HEO positively influences essential barrier proteins, particularly by robustly recovering FLG levels compromised by DNCB induction.

In further studies, we examined the effect of HEO on the protein expression of the NLRP3 inflammasome components in skin tissues using Western blotting (Figure 9D and Supplementary Materials). DNCB challenge significantly upregulated the protein expression of NLRP3 compared to the Sham group (Figure 9E, *p* < 0.001). Treatment with HEO (3%, 5%, and 10%) and Dexa significantly suppressed NLRP3 expression (*p* < 0.001), with 5% HEO showing effects comparable to the positive control. Furthermore, we assessed the activation of the inflammasome by detecting the cleavage of Caspase-1 and IL-1β. As shown in Figure 9F,G, the levels of cleaved Caspase-1 p20 and its precursor Pro-Caspase-1 were markedly elevated in the Con group. HEO treatment significantly inhibited this activation. Consistent with Caspase-1 activation, the maturation of IL-1β (cleavage of Pro-IL-1β p31 into active IL-1β p17) was significantly induced by DNCB (Figure 9H,I). HEO application effectively blocked this process, significantly decreasing the protein levels of both Pro-IL-1β and mature IL-1β p17 (*p* < 0.001). These results confirm that HEO exerts its anti-inflammatory effects in vivo by suppressing the assembly and activation of the NLRP3 inflammasome and preventing the maturation of IL-1β.

## 4. Discussion

AD is a chronic, relapsing inflammatory skin disorder characterized by skin barrier dysfunction and immune dysregulation, imposing a significant burden on patients’ quality of life [15,16]. Current therapeutic strategies, including corticosteroids and calcineurin inhibitors, often entail adverse effects that limit their long-term application, thereby necessitating the exploration of safer, natural alternatives [17,18]. This study systematically evaluated the therapeutic potential of HEO in AD. Our results demonstrate that HEO exhibits significant anti-inflammatory and barrier-repairing effects both in vitro and in vivo. In HaCaT keratinocytes and RAW264.7 macrophages, HEO effectively suppressed the LPS-induced expression of pro-inflammatory mediators, including COX-2, iNOS, NLRP3, and IL-1β, at both the transcriptional and translational levels in a concentration-dependent manner. Furthermore, HEO significantly reduced nitric oxide (NO) production in macrophages. In the DNCB-induced murine AD model, topical application of HEO markedly improved clinical symptoms (e.g., erythema, edema), inhibited ear thickening, and restored the expression of key epidermal barrier proteins, Filaggrin and Loricrin. Our findings demonstrate that HEO not only preserves cell viability but also significantly attenuates the inflammatory response, suggesting its potential as a promising therapeutic agent for managing AD-related skin inflammation.

Consistent with previous reports on the anti-inflammatory properties of *Helichrysum* species and their major constituents such as neryl acetate and β-selinene [19,20], our results showed that HEO treatment significantly suppressed the expression of key inflammatory mediators. Firstly, HEO not only inhibited the mRNA expression of iNOS and COX-2 but also significantly reduced their protein levels and the accumulation of downstream products (NO). This suggests that HEO exerts its effects via regulation at both transcriptional and post-translational levels, aligning with findings that *Helichrysum* italicum extracts can downregulate NF-κB signaling to curb iNOS/COX-2 induction [21,22]. Secondly, the significant suppression of the NLRP3 and its downstream effector IL-1β indicates that HEO may block this critical pathway to curb the inflammatory cascade. While the modulation of cytokines (TNF-α, IL-6) by *Helichrysum* oil is well-documented [23], our study provides novel evidence regarding its inhibitory effect on NLRP3 inflammasome. This finding is in line with recent studies reporting that natural products such as Esculentoside A [24] and Ginsenoside Rg3 [25] ameliorate dermatitis by modulating the NLRP3 pathway. Future in vitro studies should focus on isolating the specific bioactive components of HEO and elucidating the precise upstream signaling molecules (such as ROS and NF-κB) responsible for NLRP3 inhibition.

The anti-inflammatory effects observed in vitro were successfully validated in the in vivo AD model. HEO treatment significantly ameliorated the severity of dermatitis scores and skin lesions, exhibiting therapeutic efficacy comparable to the positive control, Dexa. This potent in vivo activity can likely be attributed to the synergistic action of HEO’s major bioactive constituents, such as neryl acetate and pinene [19,20]. Although the anti-inflammatory activity of these compounds has been reported [26], their efficacy in inhibiting ear edema and inflammatory cell infiltration in murine AD models remains unclear. Notably, HEO exhibited a non-linear concentration-response relationship, wherein the 5% concentration showed maximal suppression of ear thickness in terms mean values, without reaching statistical significance when compared to the 3% or 10% HEO groups. This response may reflect saturation of the underlying pharmacological pathway or the emergence of counter-regulatory effects mediated by constituents in HEO. This possibility need to be systematically investigated in subsequent studies. These findings highlight the importance of dose optimization in future mechanistic studies of HEO-based therapeutics.

Notably, beyond mere symptom suppression, HEO demonstrated a unique advantage in restoring skin barrier function. While DNCB induction caused a significant downregulation of FLG, HEO treatment effectively reversed this trend. The restoration of the differentiation markers is critical, as FLG degradation products are essential for maintaining skin hydration and acidity [27]. This barrier-repairing capability aligns with reports on other essential oils, such as Lavandula angustifolia [28] and Melaleuca alternifolia [29], which have been shown to accelerate barrier recovery by modulating lipid synthesis and tight junction integrity. Furthermore, compared to the long-term use of corticosteroids, which can occasionally lead to skin atrophy and barrier impairment [30], HEO offers a safer alternative that actively promotes structural repair. Therefore, the ability of HEO to upregulate the structural proteins suggests it may enhance skin defense from the root, re-establishing the physical barrier, rather than solely acting as a palliative anti-inflammatory agent.

In this study, HEO exhibited a distinct biphasic modulation on skin barrier proteins in the AD model. It significantly upregulated the reduced FLG expression triggered by DNCB administration and simultaneously suppressed the expression of LOR. This differential regulation suggests that HEO does not merely stimulate keratinocyte differentiation, but rather actively restore epidermal homeostasis. The upregulation of FLG can be attributed to the amelioration of the Th2-dominant inflammatory milieu induced by HEO. Th2 cytokines, such as IL-4 and IL-13, have been reported to directly suppress FLG gene transcription via the STAT6 signaling pathway, leading to barrier defects [31]. Although the present study did not examine the levels of Th2 cytokines, our data showed that HEO effectively inhibited the release of Th2 promoting signals such as TSLP and IL-33, indicating a role of HEO in correcting Th2 immune deviation. Thus, the suppression on FLG is terminated. Furthermore, FLG is a marker of late-stage differentiation, its restoration signifies the normalization of the keratinocyte differentiation program. Regarding the level of LOR, it has been reported that its downregulation is closely linked to the epidermal hyperplasia characteristic of DNCB-induced AD model [32]. As an early compensatory mechanism, the skin attempts to compensate the barrier damage caused by FLG deficiency by upregulating envelope proteins such as LOR [32]. In our study, we observed that the expression of LOR in the DNCB group was significantly downregulated, but the degree of this downregulation (approximately 10%) was significantly lower than that of FLG (approximately 40%). This might be an illustration of the LOR compensation effect. However, this compensatory expression of LOR is often associated with an aberrant differentiation state. The significant reduction in LOR levels following HEO treatment indicates that it suppresses the pathological epidermal hyperplasia and compensatory response driven by sustained inflammation. Thus, HEO promotes the reconstruction of epidermal homeostasis through upregulating FLG to repair the barrier and downregulating LOR to inhibit pathological compensation.

The positive drug Dexa employed in our study markedly alleviated pathological changes induced by DNCB, ameliorated systemic inflammation and liver dysfunction, inhibited the levels of the NLRP3 inflammasome in skin tissues. However, it did not exhibit restoration effects on barrier proteins such as FLG and LOR. This observation may be attributed to the complex effects of Dexa on epidermal differentiation. A previous transcriptomic profiling study by Stojadinovic et al. [33] has demonstrated that Dexa has regulatory roles at different stages of keratinocyte differentiation. Specifically, Dexa treatment alters the transcriptional landscape of primary human keratinocytes, suppressing genes involved in cell motility, wound healing, and early differentiation while inducing those associated with terminal differentiation. This biphasic effect may partly explain why Dexa displays potent anti-inflammatory activity, but failed to restore FLG and LOR expression in our model. Additionally, as a corticosteroid, prolonged exposure of Dexa has been associated with epidermal atrophy and impaired barrier recovery in both clinical and experimental settings, partly due to the suppression of keratinocyte proliferation and lipid synthesis [34]. These findings collectively suggest that Dexa effectively suppresses inflammatory cascades, its impact on the epidermal differentiation program is more complicated and may not fully recapitulate the physiological restoration of barrier proteins achieved by HEO.

Several limitations regarding the experimental model we employed should be acknowledged. The DNCB-induced mouse model is a widely accepted model for investigating chronic AD-like skin inflammation and barrier dysfunction. It should be acknowledged that the DNCB-induced model exhibits a mixed immune profile with contributions from Th1, Th2, and Th17 pathways [35,36], which differs from the predominantly Th2-skewed chronic AD in humans [35,37]. Although our current data confirms that DNCB challenge successfully upregulated key epithelial alarmins TSLP and IL-33 (Figure 7), bridging the gap to Th2 immunity, we did not perform immunophenotyping to specifically quantify infiltrating cell populations such as eosinophils, mast cells, versus neutrophils or CD4+ T cell subsets. Future studies employing more specific Th2-skewing models, such as topical MC903 (calcipotriol) application, or utilizing transgenic mice, would be valuable to further validate the specific immunomodulatory effects of HEO on the Th2 axis. Additionally, we observed significant changes in gene expression, further protein-level validation of TSLP and IL-33 via ELISA or Western blot would strengthen the mechanistic conclusions.

The safety profile of HEO is a prerequisite for its potential clinical translation. In the present study, in vitro CCK-8 assays demonstrated that HEO exhibited minimal toxicity toward keratinocytes at concentrations ≤ 20 μg/mL, establishing a safe therapeutic window for topical application. Consistent with these findings, our in vivo experiments indicated that a high concentration (5%) of HEO yielded optimal therapeutic efficacy without inducing apparent local irritation or erythema, suggesting a favorable safety margin compared to the potential atrophy associated with long-term corticosteroid use. However, to fully realize the clinical potential of HEO, several challenges must be addressed in future research: (1) Functional validation of the identified compounds and their synergism: HEO is a complex mixture of various compounds. While this study confirms the efficacy of the total oil, the specific contribution of individual constituents versus their synergistic effects remains unclear. Future studies should employ in vivo and in vitro experiments to determine the key pharmacodynamic substances that contribute to the anti-inflammasome or barrier-repairing capabilities of HEO. (2) Deepening mechanistic insights: Although we observed the reduction in NLRP3 inflammasome activation and the restoration of FLG after treatment of HEO, the upstream signaling mechanisms driving these alterations require further exploration. Future investigations should focus on whether HEO directly activates nuclear receptors involved in skin differentiation (such as PPARs) or acts indirectly by blocking the IL-4/IL-13 STAT6 signaling axis. Additionally, verifying the direct binding of HEO components to upstream targets such as TLR4 or NF-κB via molecular docking and pull-down assays would provide robust evidence for its molecular mechanism. (3) Transdermal pharmacokinetics and formulation: The high volatility and lipophilicity of essential oils pose challenges for stability and skin penetration. Future work should utilize Franz diffusion cells to quantify the retention of HEO in the epidermis versus the dermis. Furthermore, developing novel delivery systems, such as nanoemulsions, liposomes, or hydrogels, could enhance the stability of HEO, control its release rate, and improve its bioavailability in the stratum corneum. (4) Clinical translation and long-term safety: Given the anatomical differences between murine and human skin, current findings need validation in more clinically relevant models, such as Reconstructed Human Epidermis (RHE). Ultimately, randomized controlled clinical trials are necessary to evaluate the efficacy of HEO in human AD patients. Concurrently, chronic toxicity studies should be conducted to rule out potential sensitization or systemic toxicity from long-term use, ensuring its safety as a functional skincare ingredient or therapeutic agent.

## 5. Conclusions

In summary, this study demonstrates that HEO exerts potent therapeutic effects against AD by orchestrating a dual mechanism of action: suppressing cutaneous inflammation via the inhibition of the NLRP3 inflammasome/IL-1β signaling axis, and restoring epidermal homeostasis through the regulation of barrier proteins FLG and LOR. Furthermore, the favorable safety profile and lack of local irritation observed at effective dosages highlight HEO’s potential as a promising candidate for topical application. Collectively, these findings provide experimental evidence supporting HEO as a safe, natural, and effective alternative strategy for the management of AD, warranting further investigation into its clinical translation and formulation development.

## Figures and Tables

**Figure 1:**
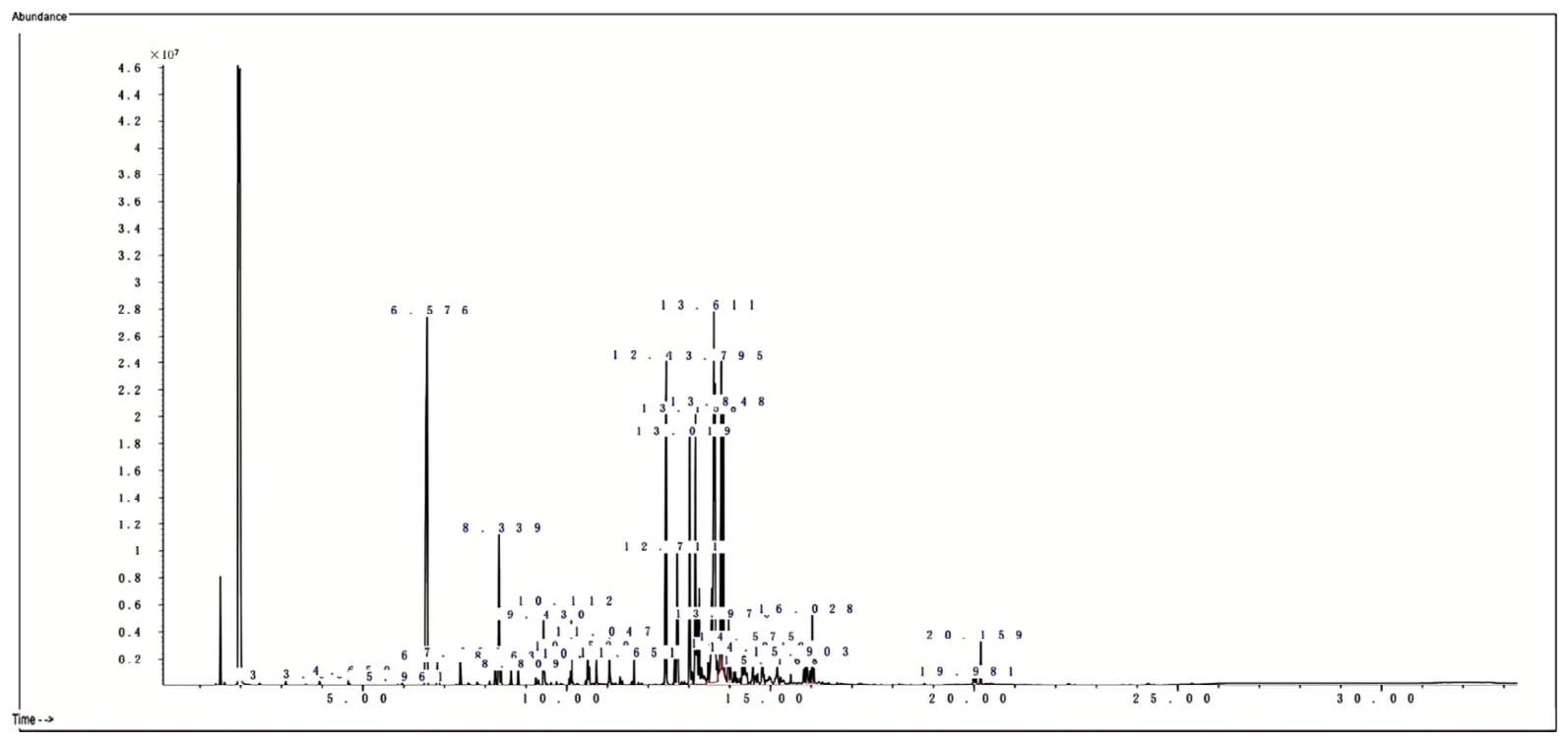
Total ion chromatogram (TIC) of HEO obtained by GC-MS analysis. The essential oil was extracted by hydrodistillation and analyzed on a non-polar capillary column. Peak numbers correspond to the compounds listed in Table 1. Major constituents include longipinene (peak 21), 1,3,3-trimethyltricyclo [2.2.1.0^2^,^6^]heptane (peak 5), β-caryophyllene (peak 20), and neryl acetate (peak 17). GC-MS conditions: electron ionization (EI) mode at 70 eV; scan range m/z 50–500; helium as carrier gas at 1.0 mL/min.

**Figure 2:**
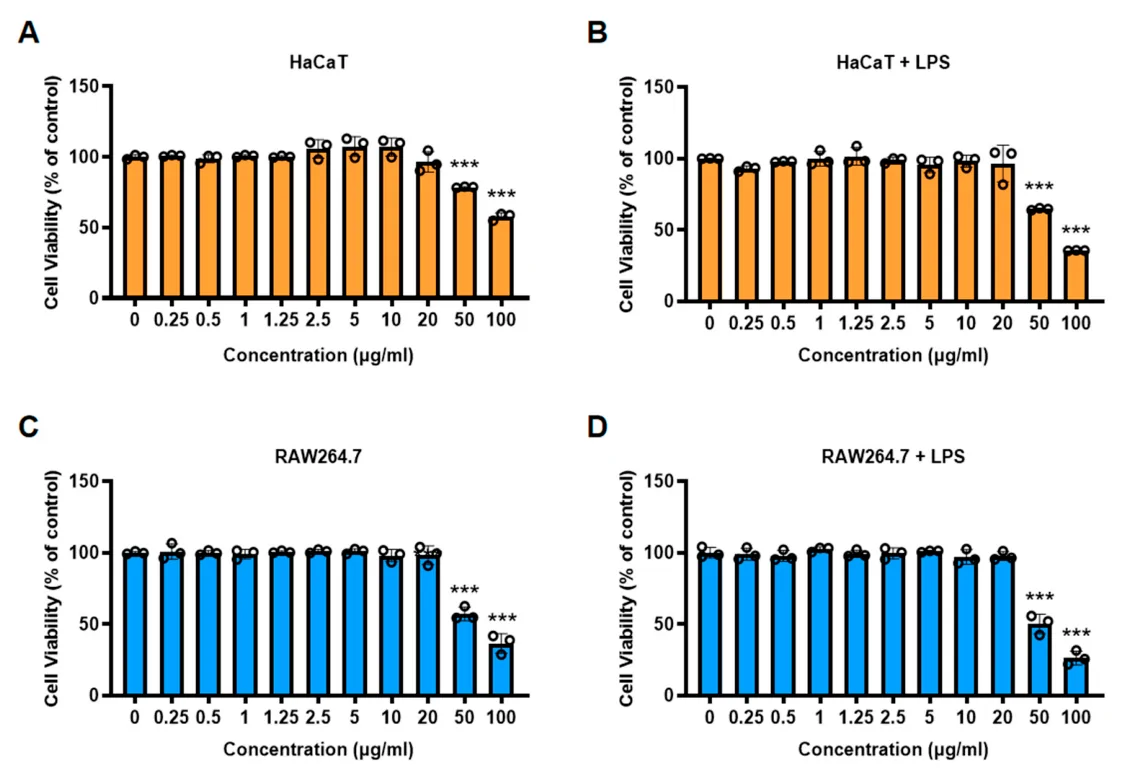
The effect of HEO on the viability of HaCaT and RAW264.7 cells. HaCaT keratinocytes (**A**,**B**) and RAW264.7 macrophages (**C**,**D**) were treated with various concentrations of HEO (0~100 μg/mL) for 24 h in the absence (**A**,**C**) or presence (**B**,**D**) of LPS. Cell viability was determined using the CCK-8 assay and is expressed as a percentage relative to the untreated control group (0 μg/mL). Circles indicate individual data points. *n* = 3 per group in (**A**–**D**). Data are presented as mean ± SD (*n* = 3). Statistical significance compared to the control group is indicated as *** *p* < 0.001.

**Figure 3:**
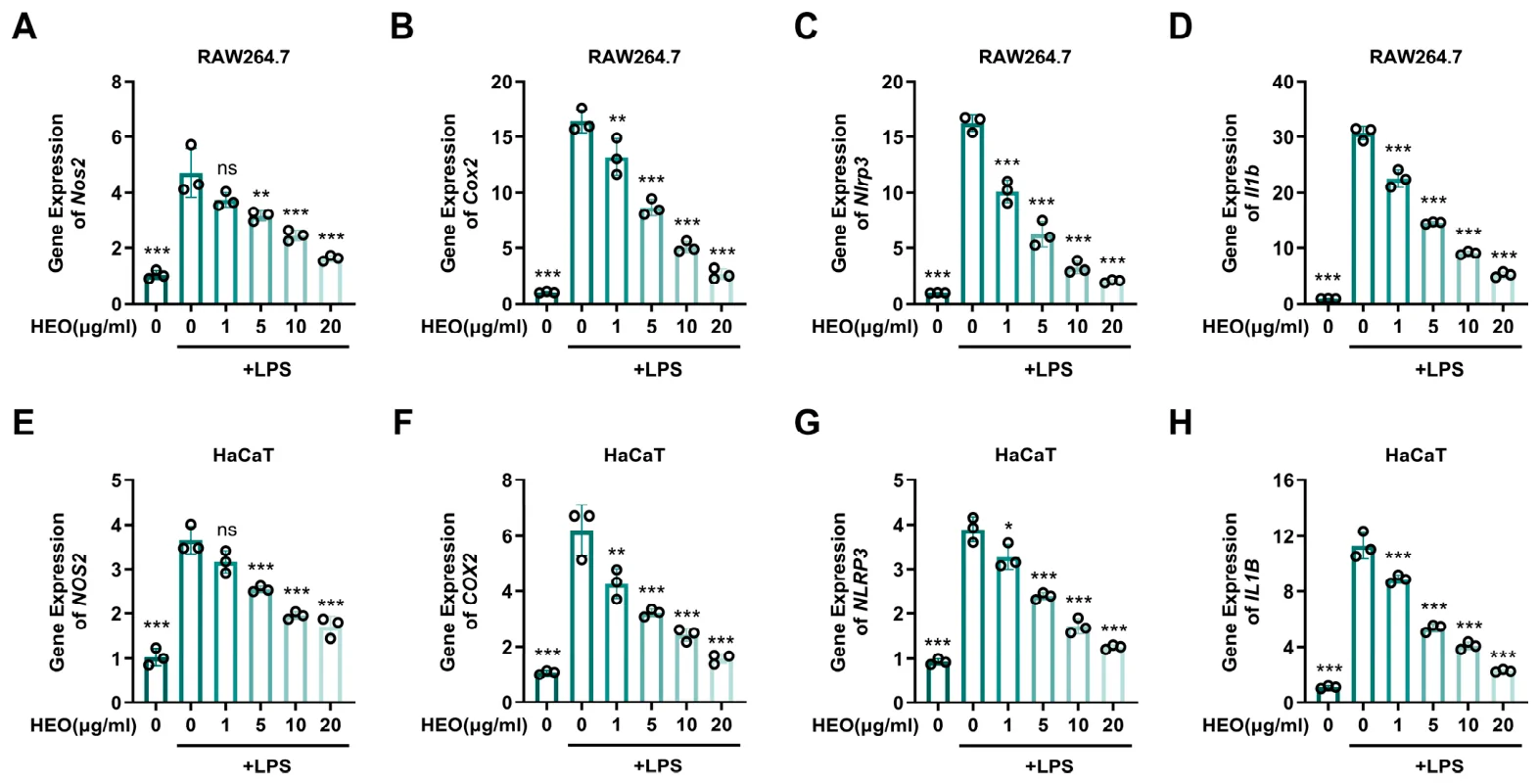
HEO suppresses the transcription of pro-inflammatory factors in RAW264.7 and HaCaT cells. RAW264.7 macrophages (**A**–**D**) and HaCaT keratinocytes (**E**–**H**) were pretreated with various concentrations of HEO (1, 5, 10, and 20 μg/mL) for 2 h, followed by stimulation with LPS for 24 h. The mRNA expression levels of iNOS (**A**,**E**), COX2 (**B**,**F**), NLRP3 (**C**,**G**), and IL-1β (**D**,**H**) were analyzed by qRT-PCR. Gene expression levels are shown as fold change relative to the unstimulated control group. Circles indicate individual data points. *n* = 3 per group in (**A**–**H**). Data represent the mean ± SD of three independent experiments. ns, not significant; * *p* < 0.05, ** *p* < 0.01, *** *p* < 0.001 vs. the LPS-only group.

**Figure 4:**
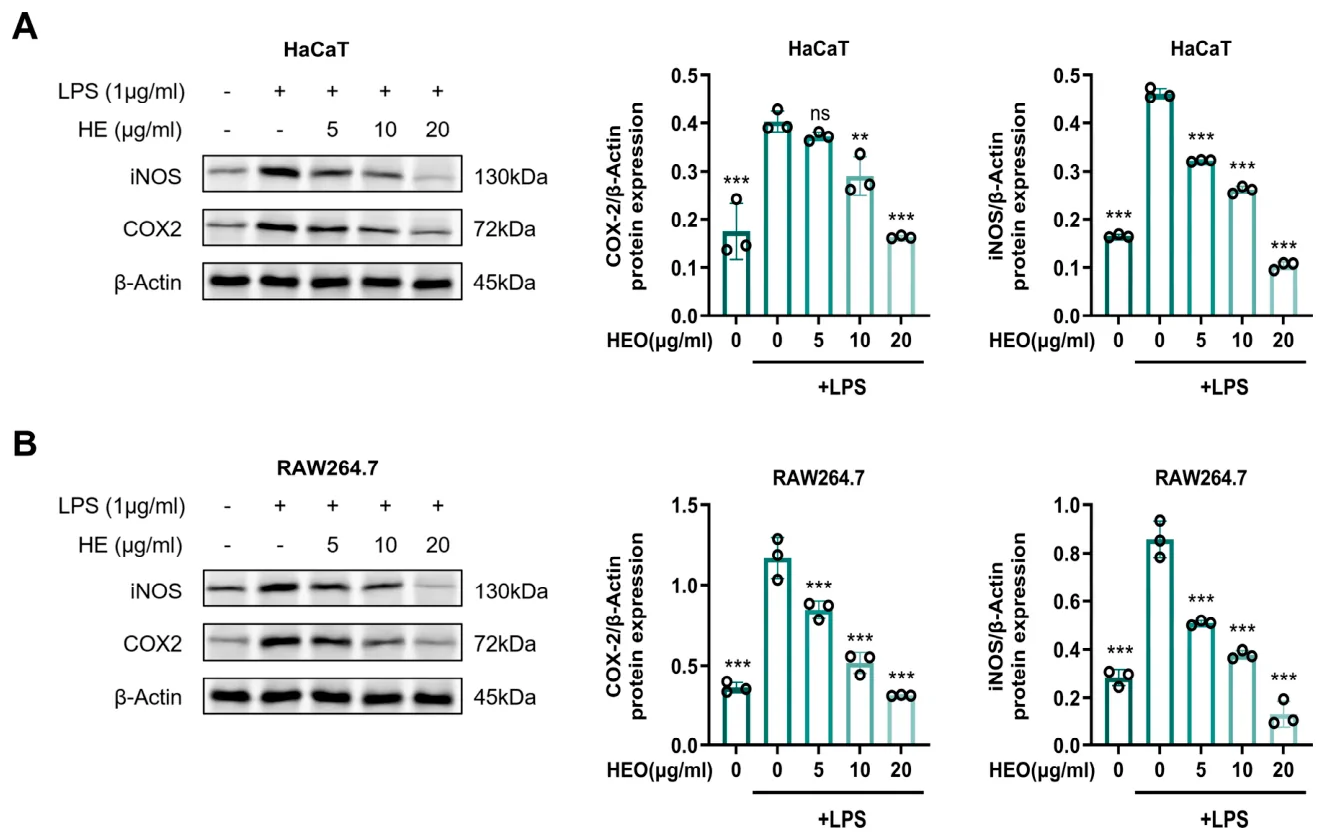
HEO significantly inhibits the expression of COX2 and iNOS in HaCaT and RAW264.7 cells. (**A**) HaCaT cells were pretreated with HEO (5, 10, and 20 μg/mL) for 2 h and then stimulated with LPS (1 μg/mL) for 24 h. The protein levels of iNOS and COX2 were determined by Western blot analysis. Quantitative analysis of the band intensities normalized to β-actin is shown in the bar graphs. (**B**) RAW264.7 cells were pretreated with HEO (5, 10, and 20 μg/mL) for 2 h followed by LPS stimulation (1 μg/mL) for 24 h. Protein expression of COX2 and iNOS was analyzed by Western blotting. Circles indicate individual data points. *n* = 3 per group in (**A**,**B**). Data are presented as mean ± SD from three independent experiments. ns, not significant, ** *p* < 0.01, *** *p* < 0.001 vs. the LPS-treated group.

**Figure 5:**
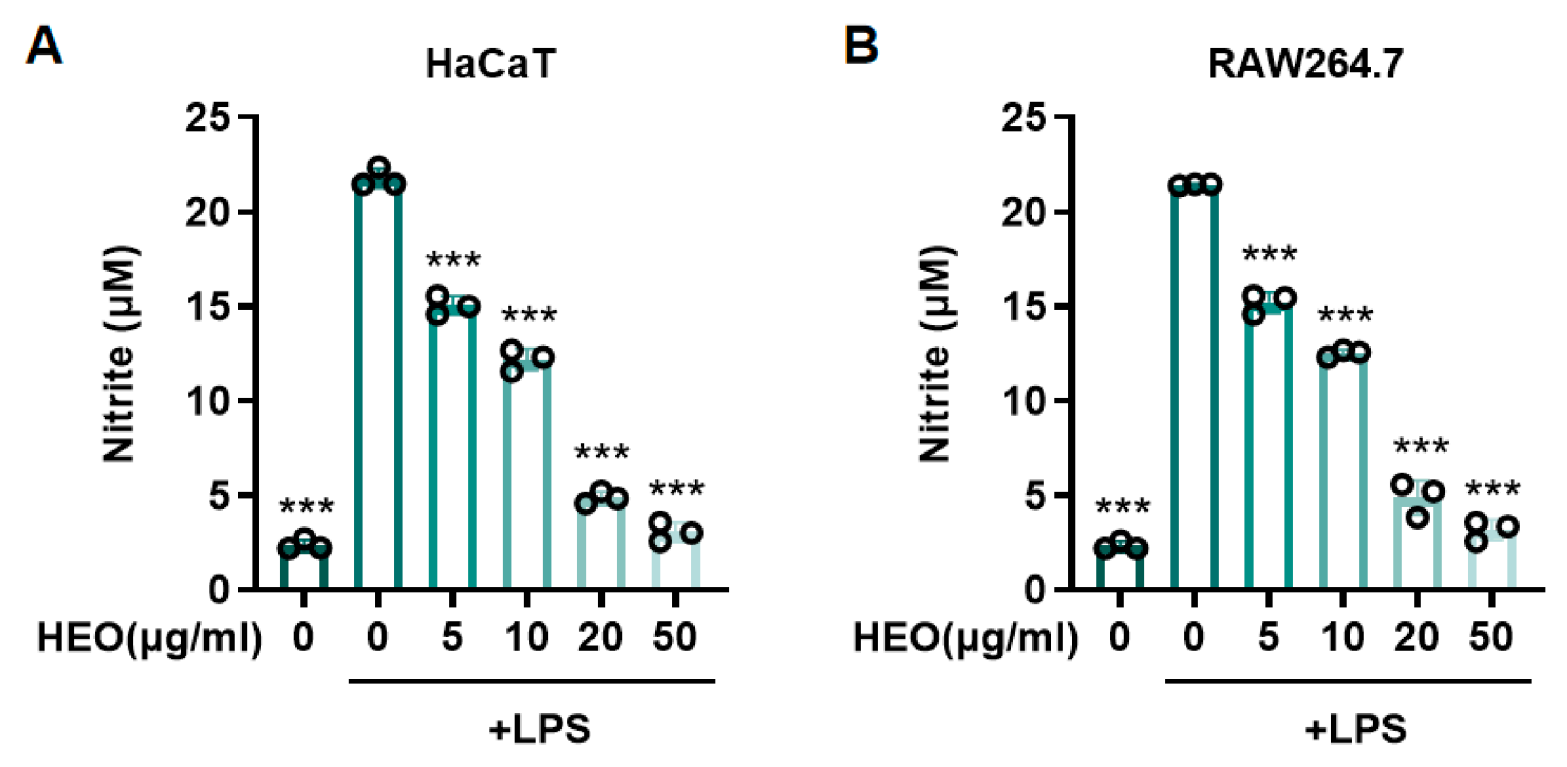
HEO suppresses NO production in LPS-stimulated HaCaT and RAW264.7 cells. (**A**) HaCaT cells and (**B**) RAW264.7 cells were pretreated with various concentrations of HEO (5, 10, 20, and 50 μg/mL) for 2 h and then stimulated with LPS (1 μg/mL) for 24 h. The accumulation of nitrite in the culture supernatants was measured using the Griess assay as an indicator of NO production. Circles indicate individual data points. *n* = 3 per group in (**A**,**B**). Data are presented as mean ± SD from three independent experiments. *** *p* < 0.001 vs. the LPS-treated group (0 μg/mL).

**Figure 6:**
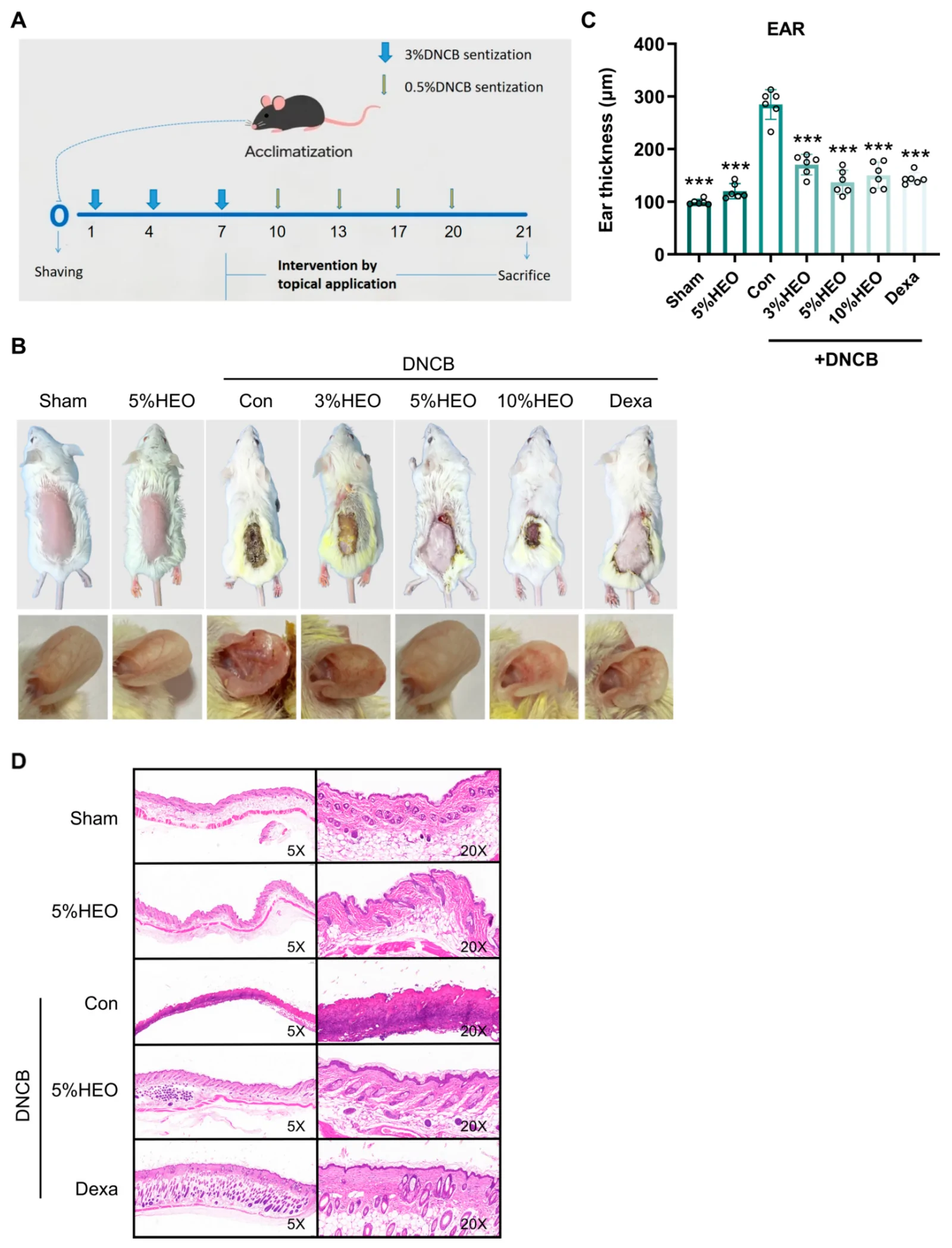
HEO significantly alleviates DNCB-induced AD in mice. (**A**) Schematic diagram of the experimental timeline for the DNCB-AD mouse model. Mice were sensitized with 1% DNCB and challenged with 0.5% DNCB. Topical treatment with HEO (3%, 5%, 10%) or Dexa was performed from day 13 to day 20, and mice were sacrificed on day 21 (*n* = 6 per group). Arrows indicate the treatment schedule. (**B**) Representative photographs of dorsal skin lesions and ears showing the therapeutic effects of HEO on AD-like symptoms. Circles indicate areas of erythema and edema. (**C**) Quantitative analysis of ear thickness (μm) in different groups (*n* = 6). Circles indicate individual data points. Data are presented as mean ± SD. *** *p* < 0.001 vs. the DNCB-treated group (Con). (**D**) Histopathological examination of dorsal skin tissues by H&E staining (*n* = 6, Scale bars: 5× and 20× magnification).

**Figure 7:**
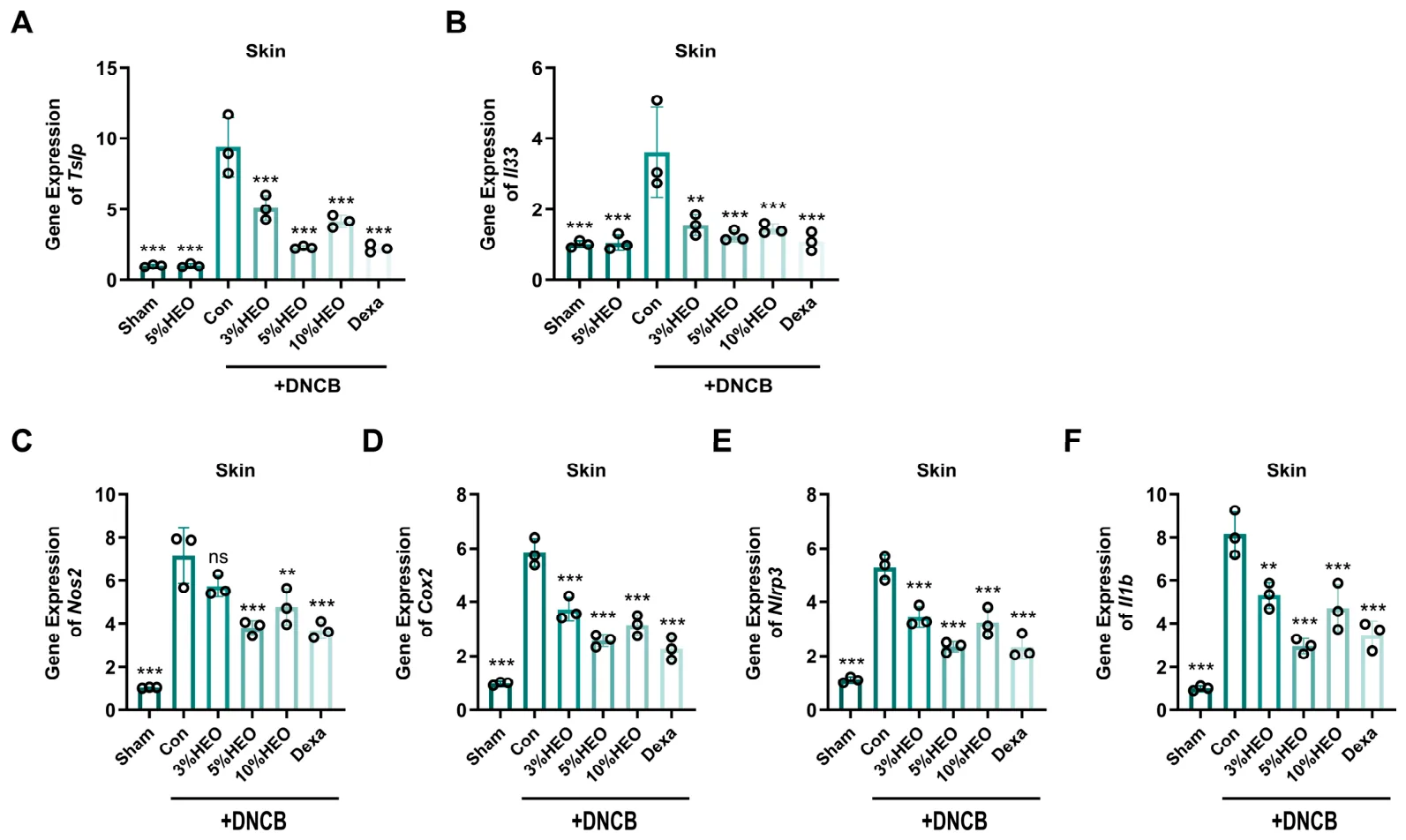
HEO suppresses the mRNA expression of inflammatory mediators in the skin lesions of DNCB-induced AD mice. Mice were treated with vehicle (Con), HEO (3%, 5%, 10%), or Dexa following DNCB induction. The relative mRNA expression levels of (**A**) TSLP, (**B**) IL-33, (**C**) iNOS, (**D**) Cox2, (**E**) NLRP3, and (**F**) IL-1β in dorsal skin tissues were determined by qRT-PCR. Circles indicate individual data points. Data are presented as mean ± SD (*n* = 3). ** *p* < 0.01, *** *p* < 0.001 vs. the Con group; ns, not significant vs. the Con group.

**Figure 8:**
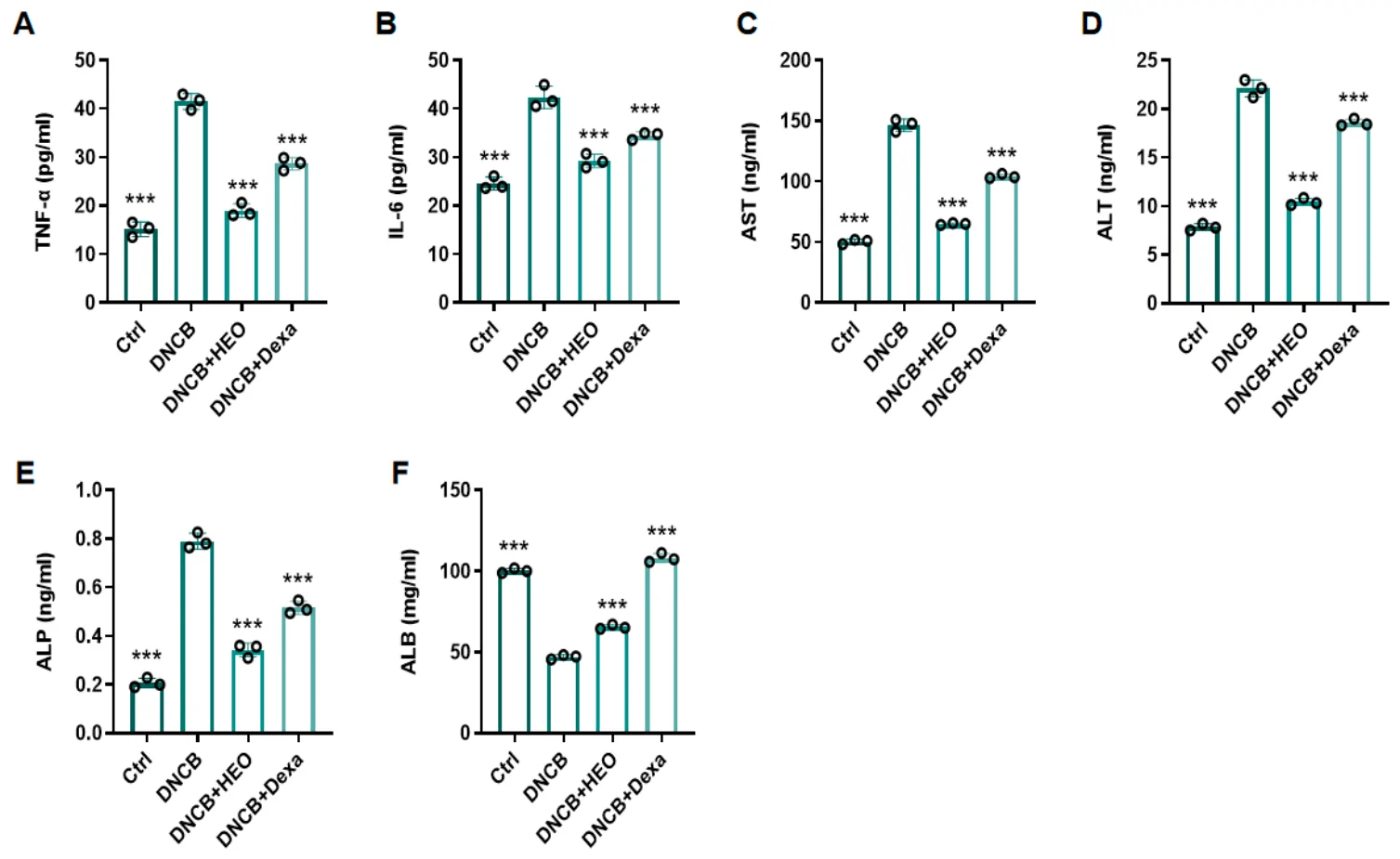
HEO ameliorates systemic inflammation and liver dysfunction in DNCB-induced mice. Serum levels of inflammatory cytokines (**A**) TNF-α and (**B**) IL-6 were measured by ELISA (*n* = 3). Liver function markers, including (**C**) AST, (**D**) ALT, (**E**) ALP, and (**F**) ALB, were analyzed in serum samples from different groups (*n* = 3). Circles indicate individual data points. Data are presented as mean ± SD. *p* < 0.001 vs. the DNCB group (or vs. Ctrl for baseline comparison). Ctrl: Control group; DNCB: DNCB-induced model group; DNCB + HEO: HEO-treated group; DNCB + Dexa: Dexa-treated positive control group. *** *p* < 0.001 vs. the DNCB group.

**Figure 9:**
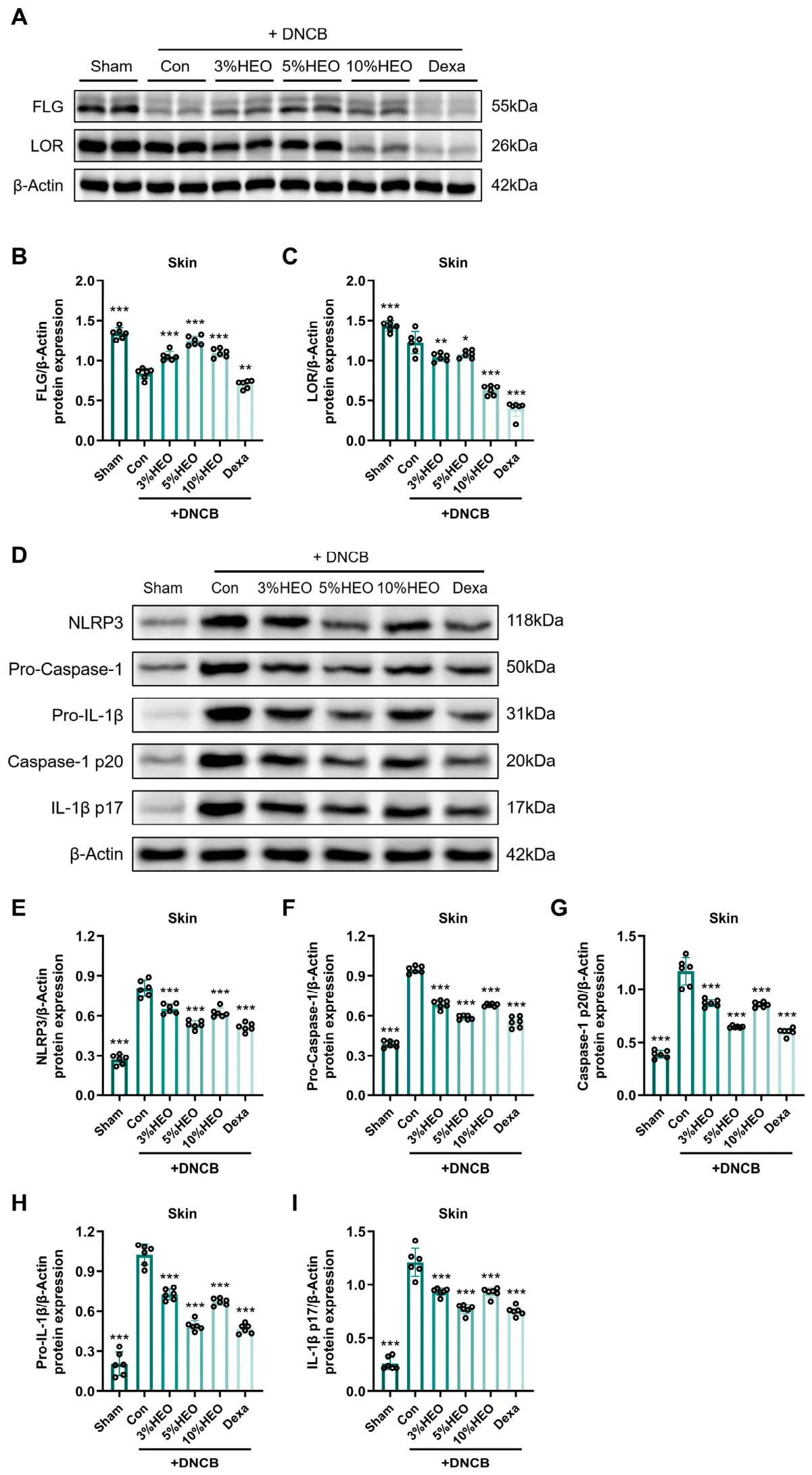
HEO restores skin barrier homeostasis and inhibits NLRP3 inflammasome activation in DNCB-induced AD mice. (**A**) Representative Western blot images of LOR and FLG in dorsal skin tissues from different groups. (**B**) Quantitative analysis of FLG protein expression normalized to β-Actin. (**C**) Quantitative analysis of LOR protein expression normalized to β-Actin. (**D**) Representative Western blot bands showing the expression of NLRP3 inflammasome-related proteins. (**E**–**I**) Quantitative analysis of NLRP3 (**E**), Pro-Caspase-1 (**F**), cleaved Caspase-1 p20 (**G**), Pro-IL-1β (**H**), and mature IL-1β p17 (**I**) protein levels normalized to β-Actin. Circles indicate individual data points. Data are presented as mean ± SD (*n* = 3). * *p* < 0.05, ** *p* < 0.01, *** *p* < 0.001 vs. the Con group.

**Table 1:** Chemical composition of HEO identified by GC-MS analysis.

No.	Compound Name	RT (min)	Area (%)
1	2-Methyl-3-pentanone	3.105	0.11
2	2-Methyl-2-heptene	3.938	0.15
3	4-Methyl-3-hexanone	4.650	0.21
4	1,7,7-Trimethylbicyclo [2.2.1]hept-2-ene	5.961	0.07
5	1,3,3-Trimethyltricyclo [2.2.1.0^2^,^6^]heptane *	6.576	15.42
6	(1R)-2,2-Dimethyl-3-methylenebicyclo [2.2.1]heptane	6.825	0.76
7	(−)-β-Pinene	7.390	0.59
8	(+)-Limonene	8.339	4.02
9	(Z)-2-Methyl-2-butenoic acid 2-methylpropyl ester	8.635	0.48
10	γ-Terpinene	8.809	0.31
11	Linalool	9.430	1.54
12	2-Methylbutyl 2-methylisocrotonate	10.112	1.67
13	3,5-Octanedione, 4,6-dimethyl-	10.520	1.21
14	α-Terpineol	10.728	0.53
15	Nerol	11.047	1.07
16	Hexyl 2-methylisocrotonate	11.651	0.56
17	(Z)-3,7-Dimethyl-2,6-octadien-1-ol acetate (Neryl acetate) *	12.442	9.35
18	Copaene (A-Copaene)	12.711	4.68
19	Cyclopenta [1,4]cyclobuta [1,2]benzene, octahydro-tetramethyl- *	13.019	5.85
20	β-Caryophyllene *	13.158	10.67
21	Longipinene *	13.611	17.60
22	β-Selinene (B-Selinene) *	13.795	7.76
23	α-Selinene *	13.848	5.37
24	Naphthalene, hexahydro-dimethyl-(1-methylethyl)-	13.975	1.87
25	3,5,7,10-Tetramethylundec-9-ene-4,6-dione	14.366	2.22
26	Caryophyllene oxide	14.575	0.92
27	(2R)-Octahydro-α,α,4aβ,8β-tetramethyl-2-naphthalenemethanol	14.812	1.10
28	(1S,8aα)-Decahydro-1,4aβ-dimethyl-7β-isopropenyl-1-naphthol	15.168	0.88
29	Humulene epoxide II †	15.903	0.62
30	(1R,4E,8E)-6,6,9-Trimethyl-2-methylene-4,8-cycloundecadiene-1-ol	16.028	1.23
31	Unidentified	19.981	0.39
32	Geranylgeraniol †	20.159	0.78
Total			99.42

* Major compounds (Area % > 5%). † Tentatively identified (NIST match < 80%). RT: retention time. Identification based on NIST 3.0 library match and retention time comparison. Bold indicates major compounds (Area % > 5%).

## Data Availability

The original contributions presented in this study are included in the article or in the Supplementary Materials. Further inquiries can be directed to the corresponding authors.

## Supplementary Materials

The following supporting information can be downloaded at: https://www.mdpi.com/article/10.3390/cimb48100996/s1.
