# Supplementary material for: Helichrysum Essential Oil Ameliorates Atopic Dermatitis by Suppressing the NLRP3 Inflammasome and Restoring Epidermal Barrier Integrity
Source: Curr Issues Mol Biol. 2026 Sep 26;48(10):996. doi: 10.3390/cimb48100996 (PMC13703321; doi:10.3390/cimb48100996)
Supplement: Supplementary file 1 [file cimb-48-00996-s001.zip › cimb-4543742-supplementary.pdf]

Figure S1A

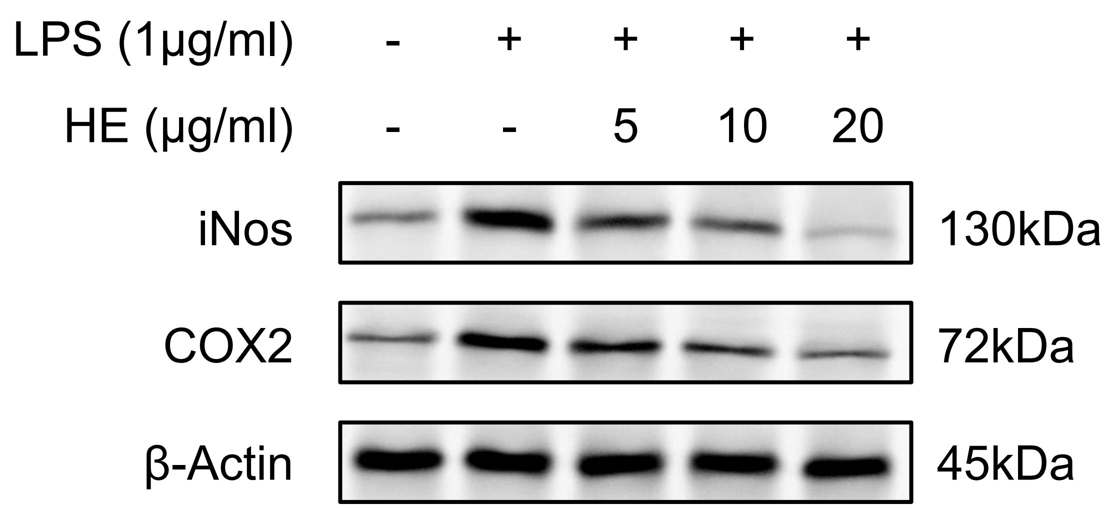

LPS (1μg/ml) - + + + +
HE (μg/ml) - - 5 10 20
iNos
130kDa
COX2
72kDa
β-Actin
45kDa

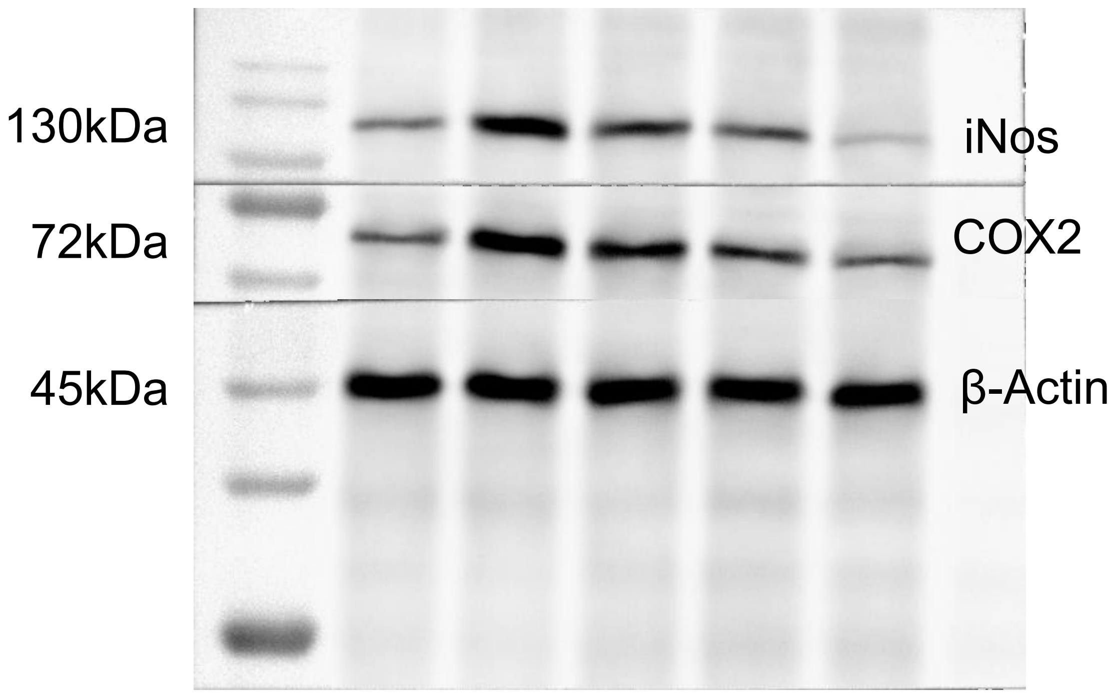

130kDa
iNos
72kDa
COX2
45kDa
β-Actin

Figure S1B

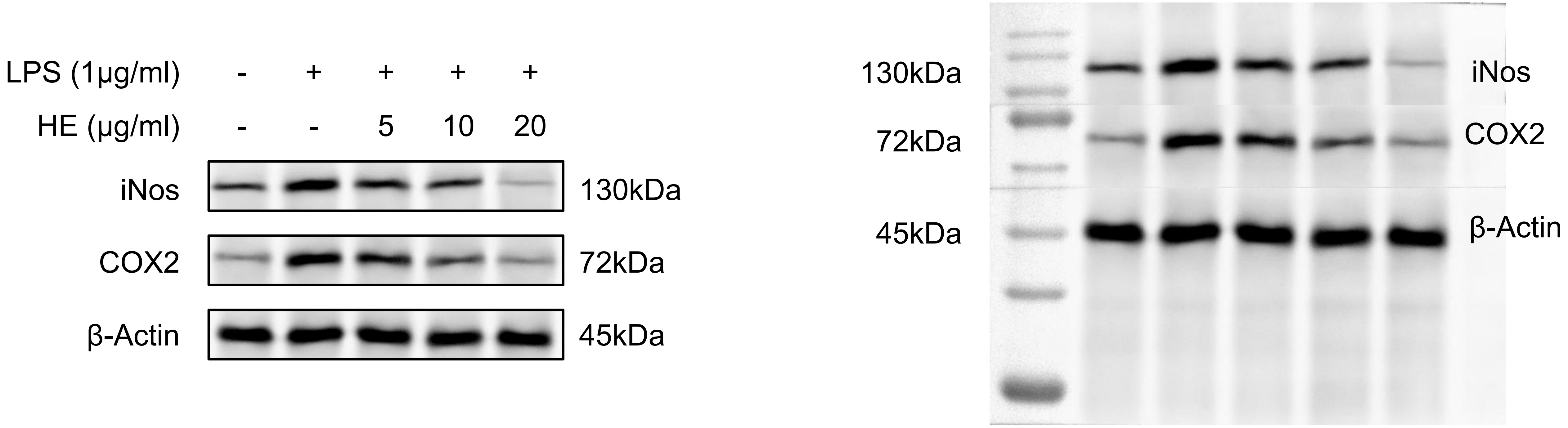
LPS (1μg/ml) - + + + +
HE (μg/ml) - - 5 10 20
iNos
130kDa
COX2
72kDa
β-Actin
45kDa
130kDa
iNos
72kDa
COX2
45kDa
β-Actin

Figure S2A

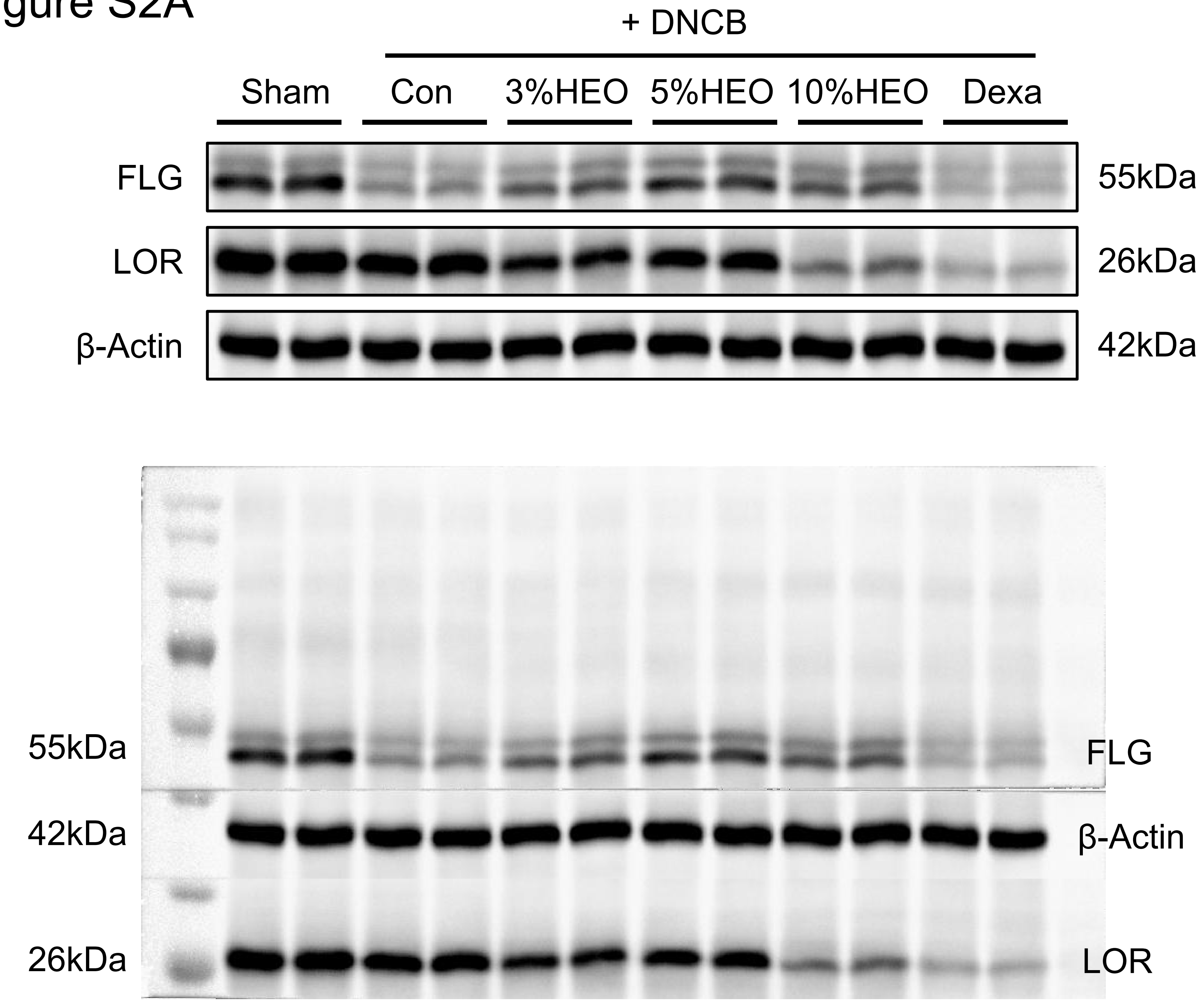
+ DNCB
Sham
Con
3%HEO
5%HEO
10%HEO
Dexa
FLG
LOR
β-Actin
55kDa
26kDa
42kDa
55kDa
42kDa
26kDa
FLG
β-Actin
LOR

Figure S2B

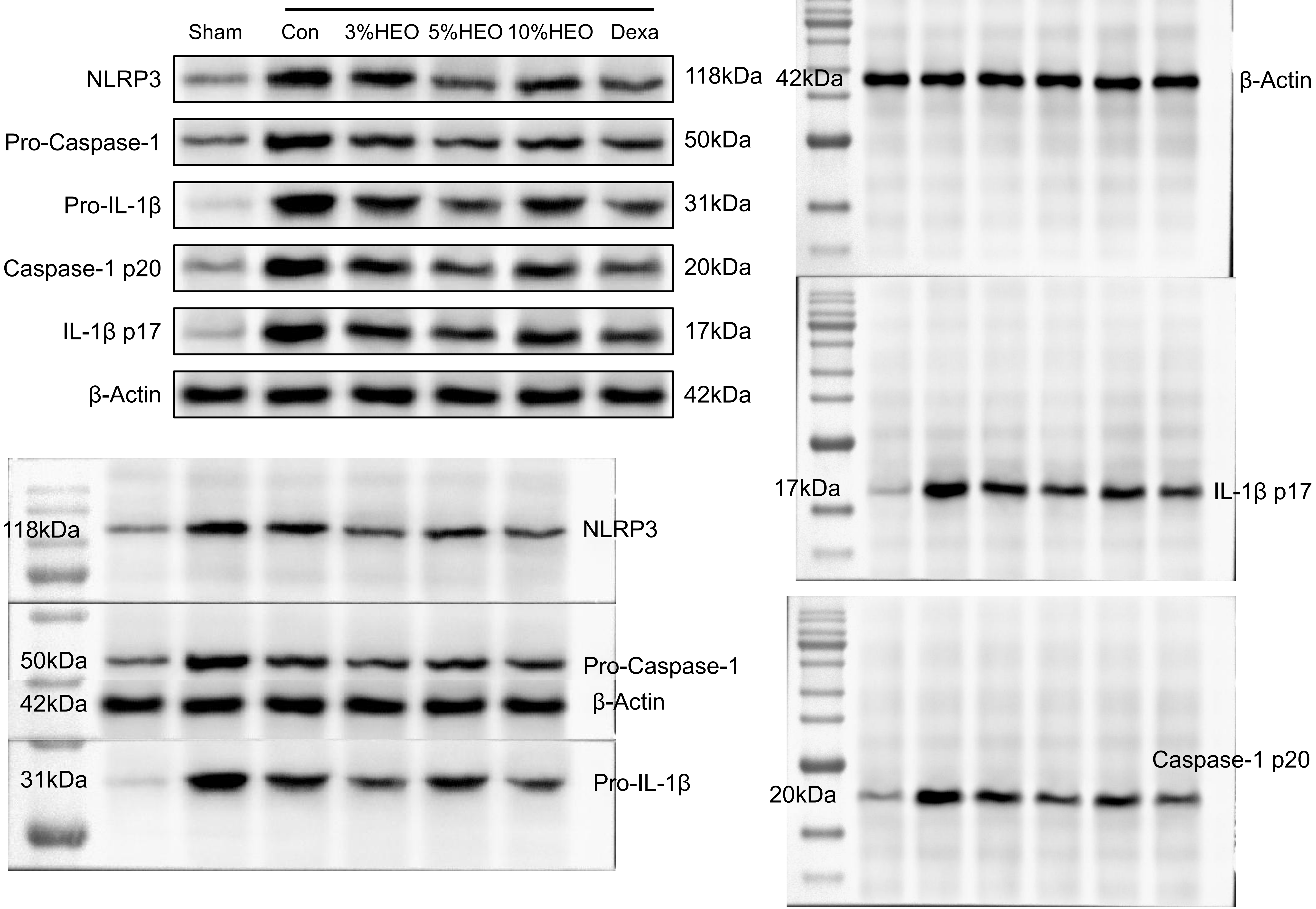
+ DNCB
Sham
Con
3%HEO
5%HEO
10%HEO
Dexa
NLRP3
118kDa
Pro-Caspase-1
50kDa
Pro-IL-1β
31kDa
Caspase-1 p20
20kDa
IL-1β p17
17kDa
β-Actin
42kDa
118kDa
NLRP3
50kDa
Pro-Caspase-1
42kDa
β-Actin
31kDa
Pro-IL-1β
42kDa
β-Actin
17kDa
IL-1β p17
Caspase-1 p20
20kDa
